# Early bone tissue aging in human auditory ossicles is accompanied by excessive hypermineralization, osteocyte death and micropetrosis

**DOI:** 10.1038/s41598-018-19803-2

**Published:** 2018-01-30

**Authors:** Tim Rolvien, Felix N. Schmidt, Petar Milovanovic, Katharina Jähn, Christoph Riedel, Sebastian Butscheidt, Klaus Püschel, Anke Jeschke, Michael Amling, Björn Busse

**Affiliations:** 1 Department of Osteology and Biomechanics, University Medical Center Hamburg-Eppendorf, Hamburg, Germany; 2 Department of Orthopaedic Surgery, University Medical Center Hamburg-Eppendorf, Hamburg, Germany; 3 Laboratory for Anthropology, Institute of Anatomy, Faculty of Medicine, University of Belgrade, Belgrade, Serbia; 4 Department of Legal Medicine, University Medical Center Hamburg-Eppendorf, Hamburg, Germany

## Abstract

Within the mineralized bone, osteocytes form a multifunctional mechanosensitive network orchestrating bone remodelling. A preserved osteocyte population is a crucial determinant of bone quality. In human auditory ossicles, the early decrease in osteocyte numbers but maintained integrity remains an unexplained phenomenon that might serve for sound transmission from air to the labyrinth. Here we analysed the frequency, size and composition of osteocyte lacunae in the auditory ossicles of 22 individuals from early postnatal period to old age. Mineralization of the bone matrix was determined using backscattered electron imaging. No signs of bone remodelling were observed above the age of 1 year. We detected characteristics of early bone tissue aging, such as decrease in osteocytes, lower total lacunar density and lacunar area, as well as high matrix mineralization accompanied by distinct accumulation of micropetrotic lacunae and decreased indentation depths. The majority of these changes took place in the first months and years of life, while afterwards only minor reorganization was present. With osteocyte apoptosis potentially being a consequence of low mechanical stimuli, the early loss of osteocytes without initiation of bone remodelling indicates an adaptive response conserving the architecture of the auditory ossicles and ensuring stable sound transmission throughout life.

## Introduction

The human auditory ossicles malleus, incus and stapes present with their final morphology at birth, while in later life decades only minor morphological changes occur^1^. The ossicles are located in the air-filled middle ear and serve for transmission of sound-induced mechanical vibrations from the eardrum to the oval window of the fluid-filled cochlea^2^. Damage or deformation of the ossicular chain lead to conductive hearing loss^3^. While malleus and incus develop from the first pharyngeal arch, the stapes which is the smallest bone in the human body has two embryologically distinct parts. In fact, the cranial end of the second pharyngeal arch forms an independent *anlage*, which develops into a superior and an inferior part. The superior part gives origin to the base of the stapes, whereas the inferior part forms the limbs (anterior and posterior crus) and the head of the stapes^4^.

As sound transmission is the major function of the auditory ossicles and biomechanical loads are minor^5^, adaptive bone remodelling might be unnecessary here. In general, the absence of bone remodelling as seen with aging would be associated with hypermineralization of the bone matrix itself, but also with the accumulation of hypermineralized (micropetrotic) osteocyte lacunae^6,7^. The latter describes the *in vivo* formation of intra-lacunar calcification^8^, which is believed to follow apoptosis of some osteocytes^7^. Hypermineralized osteocyte lacunae accumulate in aged^6^, osteoporotic^9^ and osteoarthritic^10^ bone. In the ossicles, increased numbers of dead osteocytes as a sign of impaired bone remodelling have been reported^11,12^, however the degree of mineralization of the ossicles’ bone matrix along with the distribution of viable and apoptotic osteocytes have not been shown.

We carried out this study to investigate the changes in osteocyte characteristics and matrix mineralization in the human auditory ossicles, specifically acknowledging that they are subject to unique vibrational patterns and do not experience high-strain biomechanical loading. Considering that temporal aspects of osteocyte death and subsequent hypermineralization in human ossicles are obscure, we focused on the entire age range from birth to old age. Our findings may contribute to better understanding of the osteocyte’s involvement in both bone loss pathogenesis and high bone mass syndromes.

## Results

### Morphometric and histologic characteristics

**Figure 1:**
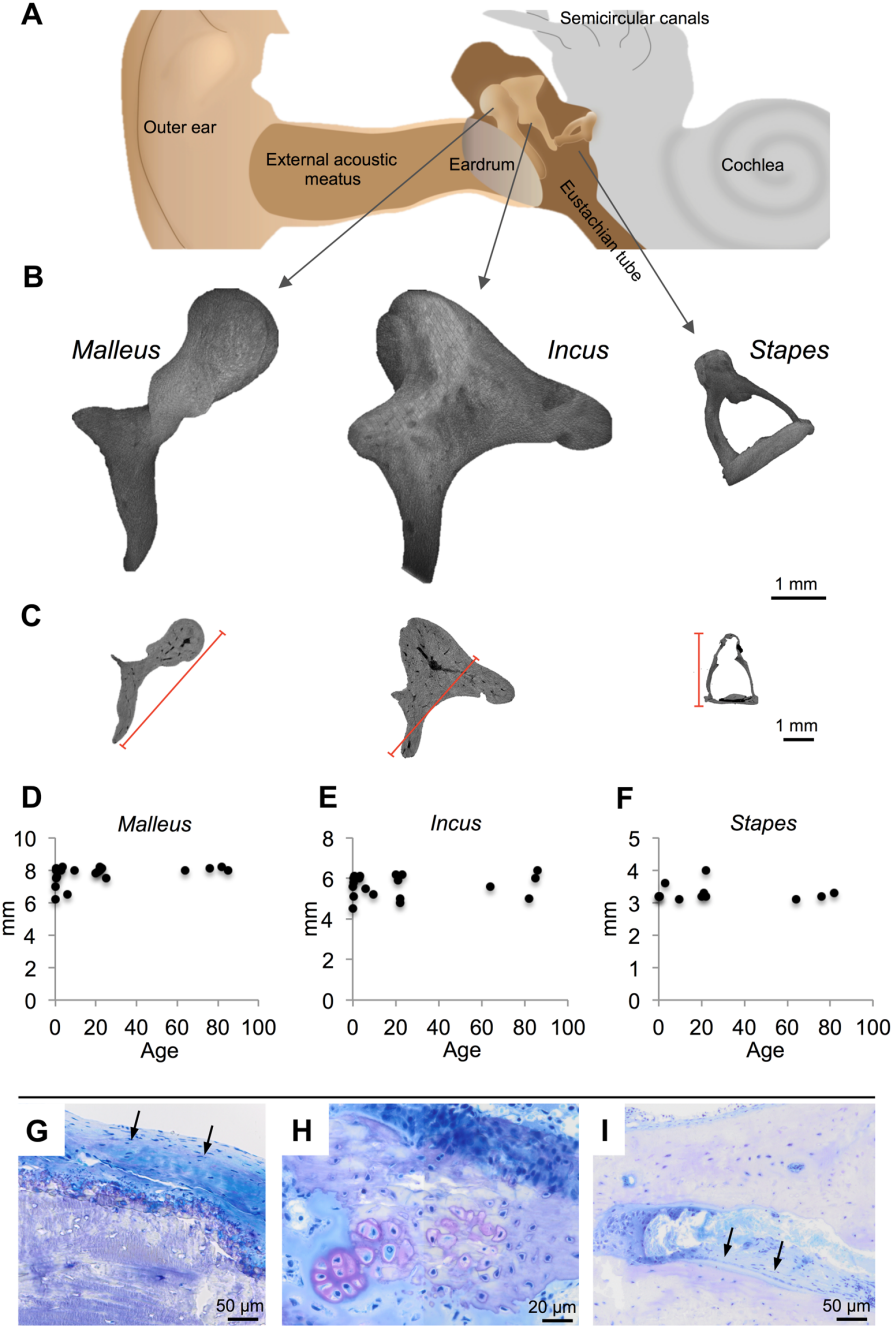
Ossicles’ dimensions and modelling. (**A**) Overview of ear anatomy with the middle ear containing the auditory ossicles. (**B**) Three-dimensional reconstructions of the malleus, incus and stapes. Images obtained by µCT imaging, resolution 6 µm. (**C**) Transverse reformats indicate a central blood vessel inside the malleus and incus. Red lines indicate the measured distances. (**D**–**F**) All three ossicles reached their final size with birth, while there was no further detectable growth with age. (**G**) A fibrous but not cartilaginous capsule surrounded the ossicle bone (malleus, black arrows). (**H**) Typical endochondral ossification with cartilaginous cells (i.e., chondrocytes). (**I**) Intramembranous ossification in the malleus of a newborn, which was only found until the age of 3 months. Black arrows indicate aligned osteoblasts on the endosteal bone surface.

The auditory ossicles analysed in this study were already completely developed at birth and had their typical shape as seen in Fig. 1A,B. A central cavity in the malleus, incus and the base of the stapes could be identified in high-resolution micro-computed tomography (µ-CT) and histology (Fig. 1B,C). Length measurements revealed that the ossicles’ size did not change after birth (Fig. 1C–F). A fibrous capsule surrounded the ossicle bone, with primary occurrence at the incumalleolar and incustapedial joint, as well as at the stapedial footplate (Fig. 1G, toluidine blue staining, black arrows). These small “joints” were not covered by typical articular (hyaline) cartilage. Only the ossicles (malleus, incus, stapes) belonging to the subjects at the age of 0 and 3 months were subject to both endochondral (Fig. 1H) and intramembranous ossification with visible osteoblasts on the endosteal bone surface (Fig. 1I). In the remaining specimens no osteoblasts or osteoclasts could be identified. In fact, no tartrate resistant acid phosphatase (TRAP) or cathepsin K-positive osteoclasts were detected in any of the biopsies.

### Mineralization and osteocyte parameters of the stapes

**Figure 2:**
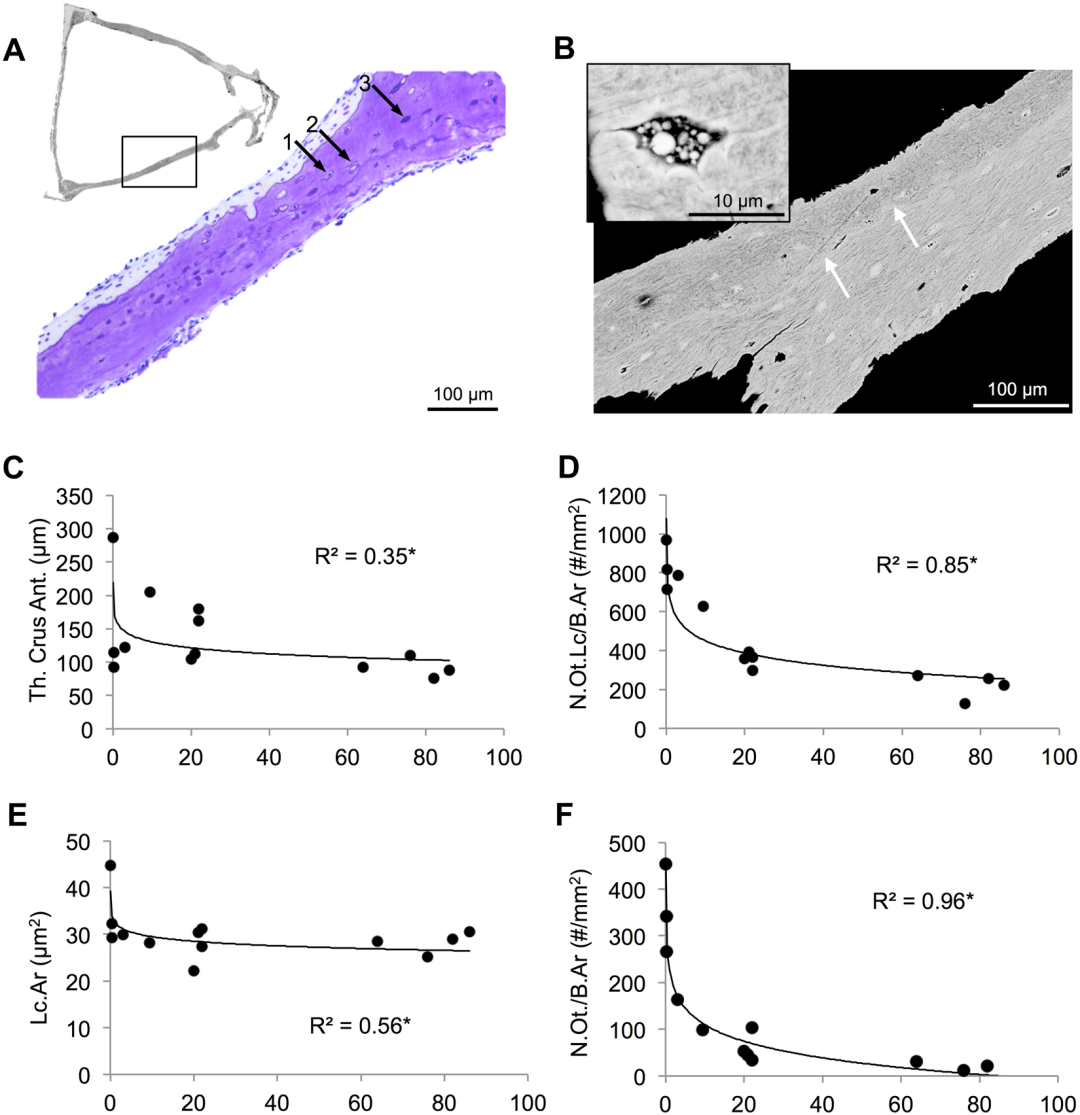
Mineralization and osteocyte parameters in the stapes. (**A**) In histological images, few viable osteocytes (with cell nucleus, 1), empty lacunae (2) and mineralized lacunae (3) were identified. (**B**) In corresponding quantitative backscattered electron microscopy, these lacunae (3) appeared highly mineralized (micropetrotic). Higher resolution imaging revealed that mineralized lacunae often contained calcified nanospherites (box). A mineralized cement line (white arrows) was seen in all specimens indicating the boundary between the two embryonic origins. (**C**) The thickness (Th) of the crus was high at birth, however already reduced in the next months. (**D**) The number of osteocyte lacunae as quantified by *qBEI* decreased with age. (**E**) After reduction in the first year, lacunar area (Lc.Ar) reached an early plateau phase. (**F**) The number of osteocytes (N.Ot) per bone area (B.Ar), quantified as lacunae with cell nucleus by histology, showed a highly significant reduction with age. *p < 0.05.

In the histological analyses, as well as in quantitative backscattered electron imaging (*qBEI*) of the stapes, we detected a high number of both empty and hypermineralized (micropetrotic) osteocyte lacunae from very young age (Fig. 2A,B, Supp. Figure 1). Some of the micropetrotic lacunae were not homogenously filled with mineralized material, but rather showed a heterogeneous accumulation of calcified nanospherites (Fig. 2B). A highly mineralized line (i.e., “cement line”) was seen as a boundary between the internal and external portion of the crura of the stapes, indicating that they have two distinct embryological parts (i.e., superior, inferior) that arise from a separate *anlage* (Fig. 2B, white arrows).

**Figure 3:**
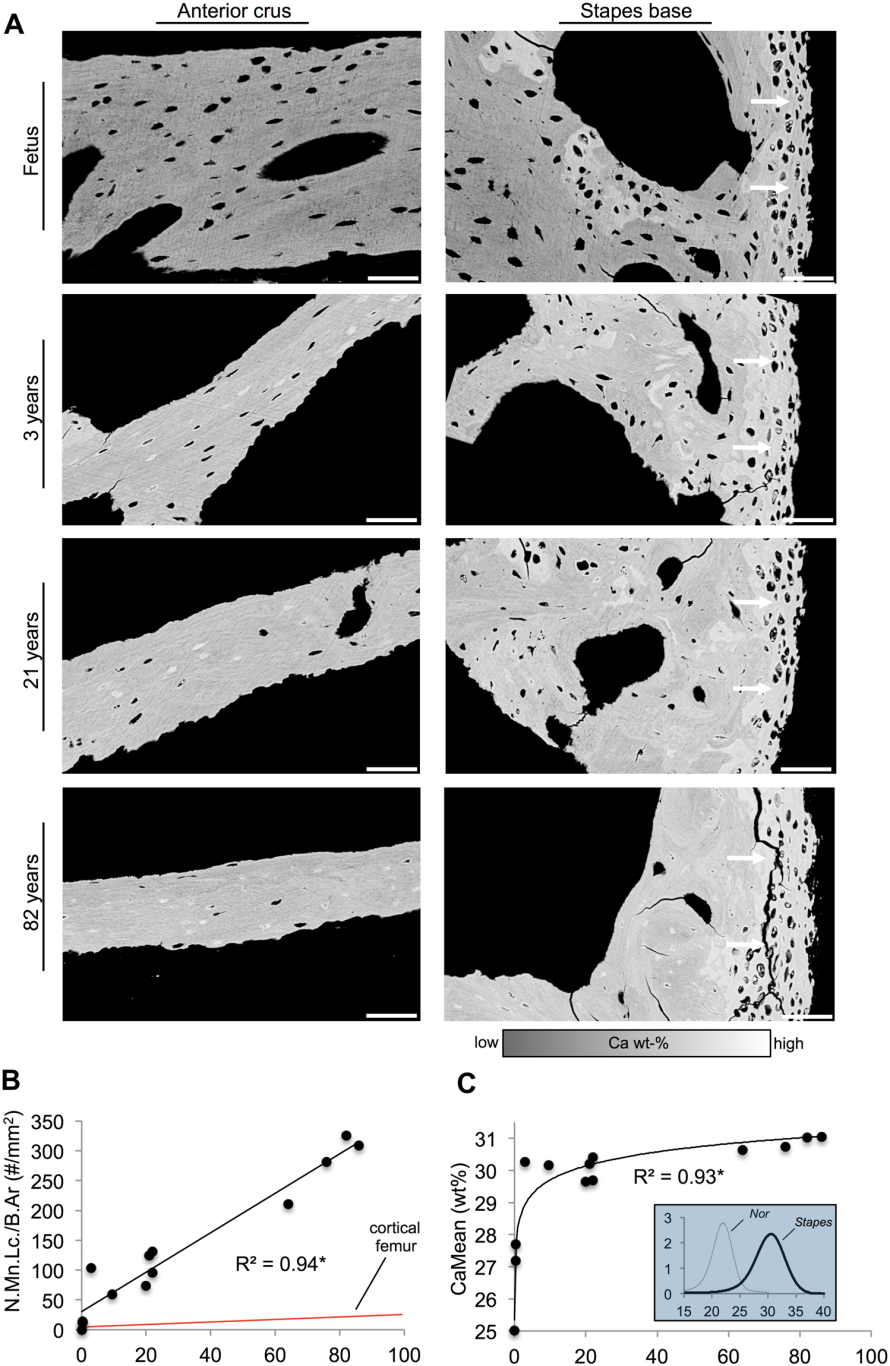
High bone mineralization and micropetrosis in the stapes. (**A**) Note the high mineralization and formation of hypermineralized lacunae at early life stages. A highly mineralized layer with cartilaginous cells covered the stapedial footplate (white arrows). Scale bar 100 µm. (**B**) The number of mineralized lacunae (N.Mn.Lc/B.Ar) showed a linear increase with age, which was much more prominent than in control from femur cortical bone (red line)^9^. (**C**) Mean calcium content (CaMean) increased with age leading to a highly mineralized bone matrix in the first years of life. *p < 0.05.

There was an age-dependent trend of decreasing thickness of the anterior crus of the stapes; the most dramatic thinning occurred already during the first year and only minor changes occurring afterwards (logarithmic regression: r^2^ = 0.351, p = 0.033) (Figs 2C, 3). Importantly, we could demonstrate a significant age-related decline in osteocyte numbers. Specifically, number of osteocyte lacunae per bone area showed an age-dependent decline that was best and highly explained by logarithmic fitting (r^2^ = 0.854, p < 0.001), considering that the most dramatic reduction occurred during the first postnatal months followed by a more gradual reduction (Fig. 2D). Lacunar area (Fig. 2E) also demonstrated a reduction with age that was most prominent during the first year (logarithmic fit: r^2^ = 0.559, p = 0.003). The number of viable osteocytes per bone area as assessed by histology decreased in an analogous manner (logarithmic fit: r^2^ = 0.964, p < 0.001; Fig. 2F), so that lacunar occupancy also decreased (logarithmic fit: r^2^ = 0.774, p < 0.001, not shown). Backscattered electron imaging (Fig. 3) revealed that mineralized osteocyte lacunae were present already at birth, but unlike other osteocyte related parameters, their number showed a linear increase with age (r^2^ = 0.941, p < 0.001) (Fig. 3B). Backscattered electron imaging of healthy femoral diaphyseal biopsies revealed a considerably lower increase in the number of micropetrotic lacunae with aging (Fig. 3B- red line^9^).

Quantitative backscattered electron imaging revealed a high bone matrix mineralization (Fig. 3). Calcium weight percentage values increased with age remarkably during the first three years, followed by minor changes after that age (logarithmic fit: r^2^ = 0.925, p < 0.001; Fig. 3C).

Further correlation analysis showed that an increase in Calcium mean values (Ca mean) correlates significantly with accumulation of mineralized lacunae per bone area (positively) and negatively with other parameters (crus thickness, osteocyte lacunae, lacunar area, osteocyte number, lacunar occupancy).

### Mineralization and osteocyte parameters of the malleus

**Figure 4:**
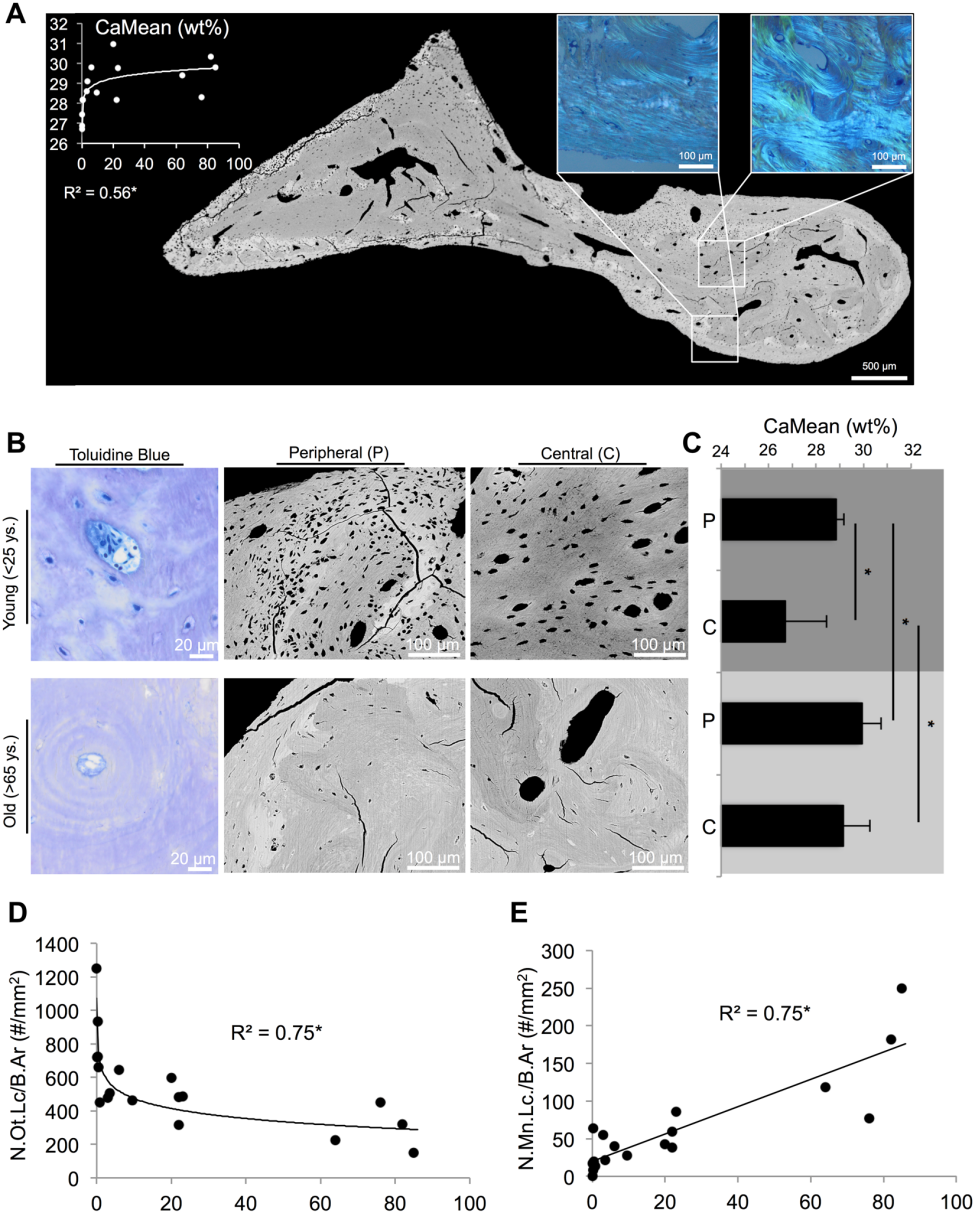
Osteocyte and mineralization characteristics in the malleus. (**A**) CaMean increased logarithmically with age. Backscattered electron microscopy (BSEM) of one complete malleus shows a highly mineralized frame. White boxes: polarized light microscopy indicated the disorientated collagen structure in the peripheral parts when compared with the central parts. (**B**) Toluidine blue histology and BSEM of young and aged specimens. Note the decay of osteonal structures, osteocyte lacunae and age-related adjustment of mean calcium content between central and peripheral parts. (**C**) *qBEI* revealed a significant difference between young and aged specimens regarding CaMean, and a significant difference between central (C) and peripheral (P) parts (especially in young cases). (**D**,**E**) Age-related reduction in the number of osteocyte lacunae (N.Ot.Lc) and increase in number of mineralized lacunae (N.Mn.Lc/B.Ar) in the malleus, which show resemblance with the findings in the stapes.

Quantitative backscattered electron imaging showed a significant trend of increasing mineral content of the bone matrix with age (logarithmic fit: r^2^ = 0.559, p = 0.001; Fig. 4A). Examination with high magnification revealed that malleus had a frame of highly mineralized bone tissue in the peripheral zones (Fig. 4A). These zones showed disorientated collagen orientation as seen by polarized light microsopy (Fig. 4A, insets). Furthermore, the peripheral zones of the malleus were significantly higher mineralized than central zones adjacent to the central blood vessel (p = 0.029) (Fig. 4B,C). While both the peripheral and central zones showed a significantly increased mineral content in aged cases (peripheral p = 0.014; central p = 0.046), the mineralization discrepancy between the peripheral and central zones showed a tendency to decline (Fig. 4B,C).

Viable osteocytes were highly frequent in young cases compared to older cases, where almost no osteocytes were found in the remaining non-mineralized lacunae (Fig. 4B, toluidine blue staining). Regression analyses revealed a logarithmic decline in osteocyte lacunar number (r^2^ = 0.752, p < 0.001; Fig. 4D) as well as osteocyte lacunar size (r^2^ = 0.779, p < 0.001, not shown) as a function of age. Number of mineralized lacunae increased with age in a linear manner (r^2^ = 0.753, p < 0.001, Fig. 4E).

### Canalicular connections and osteocyte apoptosis

**Figure 5:**
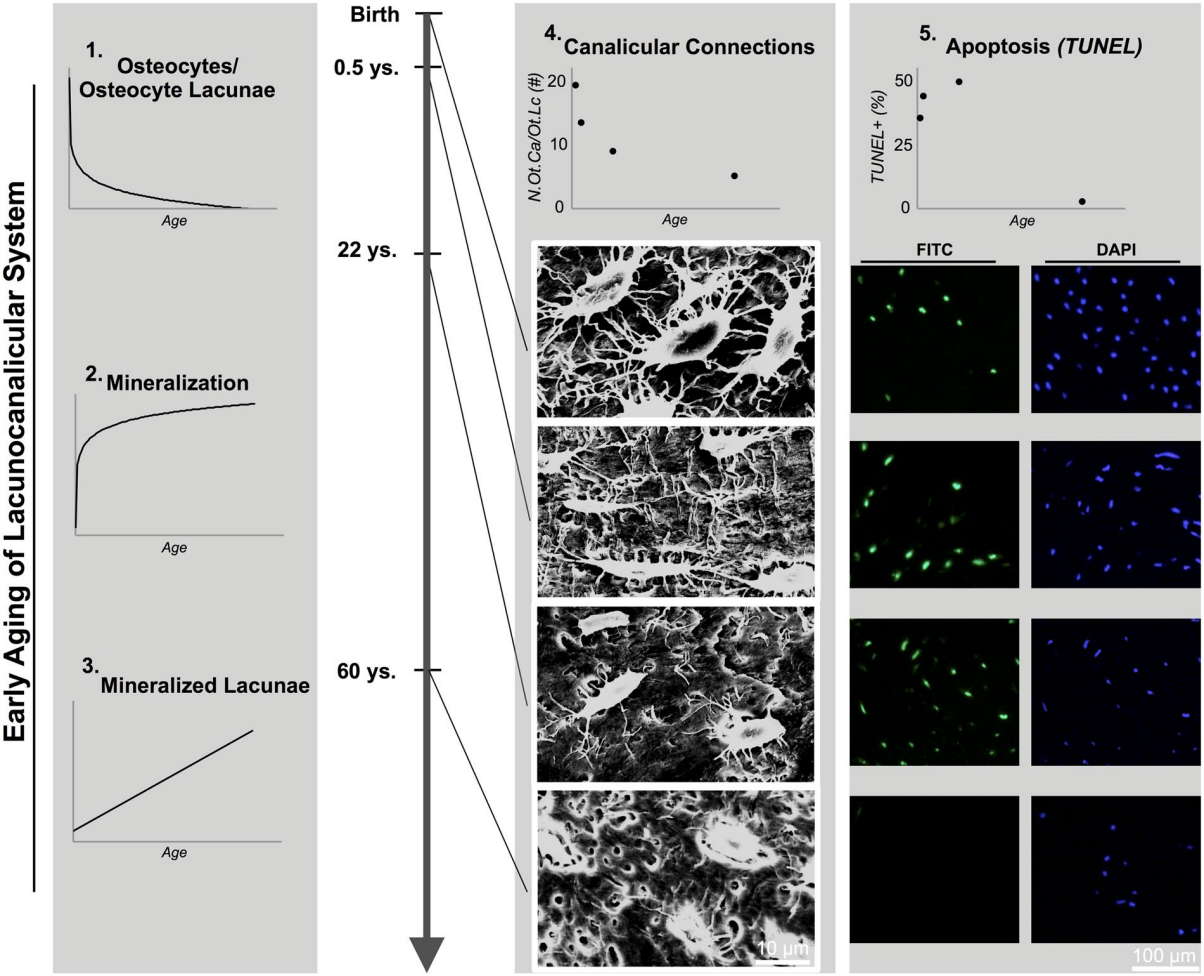
Osteocyte canalicular connections and apoptosis. (1.–3.) Diagrams summarizing the age-related changes in osteocyte number, mineralization and number of mineralized lacunae in the ossicles. (4.) Acid etching of plastic embedded bone specimens revealed a reduction in number of canaliculi per lacuna, as well as fewer connections between osteocyte lacunae with age (N.Ot.Ca/Ot.Lc). (5.) TUNEL positive (apoptotic) cells were quantified and revealed a maximum at the age of 22 years.

Next to the detected age-related trends in osteocyte lacunar number, mineralization and micropetrosis (Fig. 5; 1.–3.), acid etching and subsequent scanning electron microscopy revealed a high connectivity among osteocytes (i.e., number of osteocyte canaliculi per osteocyte lacuna) during the first year of life, in comparison to poorly connected osteocyte lacunae evident later during lifetime (Fig. 5, 4.). Equally, the mean canalicular length declined from 16.2 ± 6.8 µm at birth to 13.3 ± 4.2, 7.9 ± 2.4 and 6.5 ± 3.1 µm at 0.5, 22 and 60 years.

Terminal deoxynucleotidyl transferase dUTP nick end labelling (TUNEL staining) allowed us to detect the amount of osteocytes undergoing apoptosis related to the total cell number (Fig. 5; 5., Supp. Figure 2), confirming a highly dynamic osteocyte apoptosis process that occurs at an early age leading to large numbers of dying osteocytes. Apoptotic nuclear fragmentation and nuclear blebbing were identified by TUNEL staining procedure under FITC (Fluorescein isothiocyanate) and DAPI (4′,6-diamidino-2-phenylindole) filters (Supp. Figure 2 C, D). The relative amount of osteocyte apoptosis increased until to age of 22 years, while at the age of 60 years we found essentially no osteocytes undergoing apoptosis (Fig. 5; 5.).

### Reference point indentation of mallei

**Figure 6:**
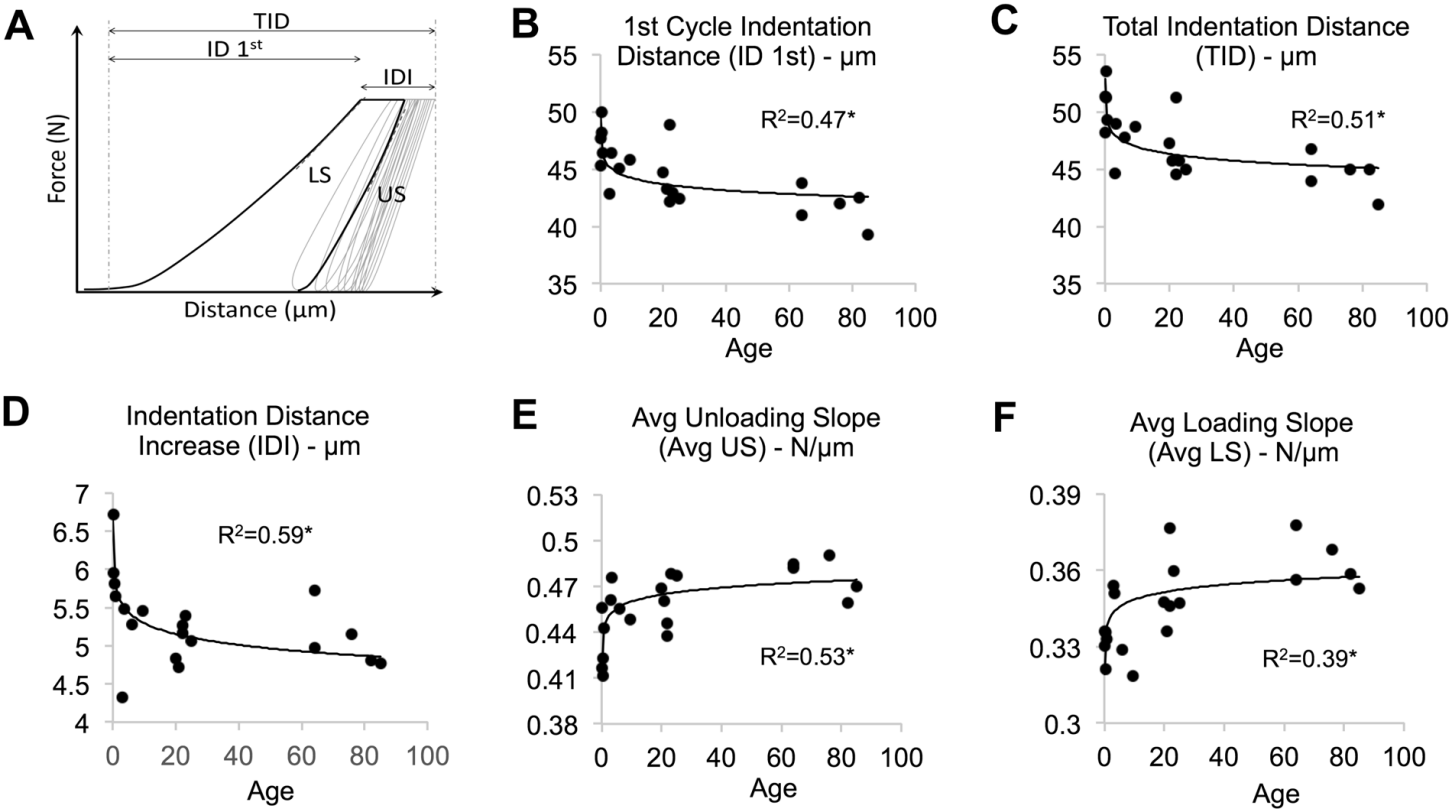
Biomechanical testing of the mallei using reference point indentation. (**A**) The load-displacement curve that is taken during microindentation facilitates the quantification of RPI parameters. (**B**) First cycle indentation distance (ID 1^st^, µm). (**C**) Total indentation distance (TID, µm). (**D**) Indentation distance increase (IDI, µm). (**E**) Average unloading slope (Avg US, N/µm) and (**F**) average loading slope (Avg LS, N/µm).

Reference point indentation of the mallei allowed the extraction of parameters indicating local bone material properties (Fig. 6A). A significant age-dependent decline in the first cycle indentation distance (ID 1^st^) was detected (logarithmic fit, r² = 0.47, p < 0.01) indicating a higher resistance to deformation with increasing age (Fig. 6B). Consistently the total indentation distance (TID) (Fig. 6C) decreased with age (r² = 0.505, p < 0.001) supporting the ID 1^st^ data. The indentation distance increase (IDI) (Fig. 6D) appeared to decrease with age (p < 0.001, r² = 0.594). The average unloading slope (Avg US) and average loading slope (Avg LS) (Fig. 6E,F) increased with an increasing tissue age (p < 0.001, r² = 0.525 and p < 0.01, r = 0.391 respectively). Higher Avg US suggests an increasing stiffness of the bone material with age.

## Discussion

The natural lifespan of osteocytes is believed to be about 25 years^13^. Our results in the auditory ossicles show a dramatic decrease in viable osteocytes already in early childhood, which indicates that the lifespan of osteocytes might be highly site-specific.

Our results indicate that high-frequency vibration in auditory ossicles goes along with osteocyte apoptosis. It is generally known that viable osteocytes provide anti-resorptive signals, while osteocyte apoptosis (such as due to dendrite transection by a microcrack) normally attracts osteoclasts to resorb the bone at the site^14,15^. However, the pronounced osteocyte death and subsequent massive accumulation of dead osteocytes without increased bone resorption suggest that there is a factor inhibiting bone resorption in the ossicles, which could be of major interest for the treatment of human bone loss syndromes i.e., osteoporosis. Another reason for the absent bone resorption could be that the osteocyte-derived proteins triggering bone resorption such as RANKL^16,17^ cannot get to the bone surface due to an early decline in the number, length and size of osteocyte canaliculi. While further research is necessary to reveal the exact nature of an osteoclasts-inhibiting signal, such signal could be perceived as a factor conserving the overall architecture of the ossicles to maintain optimal sound transmission. The subsequent excessive formation of hypermineralized lacunae represents a unique feature in auditory ossicles, as no other skeletal site (e.g. human cortical bone, iliac crest biopsies^6,10,18^) has shown micropetrotic lacunae in this order of magnitude.

Recently, it was shown that nanospherites can grow and fuse until complete occlusion of the lacuna^19^. Such nanospherites were also frequently found within various osteocyte lacunae in the analysed ossicles. Along with osteocyte apoptosis, nanospherite formation and micropetrosis, the ossicle’s bone matrix becomes very highly mineralized during the first years of age with calcium weight percentages of up to 31 wt%. These are the highest reported levels for mean mineralization in the human skeleton in comparison to weight percentages of 22 to 25 wt% in human cortical or trabecular bone^9,20,21^. Interestingly, the mean calcium content in the ossicles increased logarithmically with age, while the number of micropetrotic lacunae increased linearly, which shows that matrix hypermineralization and formation of micropetrotic lacunae are not directly related or occur in a time-delayed manner.

Both mineralization and aging are known to influence the bone’s material properties^22,23^. We have conducted reference point indentation on the mallei to find out whether the mechanical properties change comparably to age-related changes in mineralization pattern and osteocyte parameters. Our data suggest that the resistance to deformation (i.e., ID 1^st^, TID) of ossicles in infants (<1 year) corresponds to that of cortical bone in individuals of around 52 years of age^24^. Furthermore, our results strongly underline that the age-dependent changes in auditory ossicles do not only include highly dynamic changes in osteocyte number and mineralization but also an increasing resistance as testified by decreasing indentation depths. Furthermore, early age-related increases in the average unloading slope suggest stiffening of the material at higher mineralization levels^25^. The indentation distance increase (IDI), which can be interpreted a measure of plasticity^26^, revealed a significant, logarithmic decrease with aging, which may seem contradictory to previous findings^27^. However, it was also shown that a decrease in IDI is linked to increasing mineral content and aging in early stage, which is indeed the case in the ossicles^25^. In fact, the IDI reached an early plateau phase and no significant changes took place after the age of 1 year. Since all parameters extracted from the RPI load-displacement curves followed a similar development during aging as osteocyte parameters and overall matrix mineralization, it can be proposed that both have a major influence on the mechanical properties of bone. These mechanical properties reflecting a stiff and hard material may be physiologically ideal to transfer the vibrations of sound with losing as little energy as possible.

In general, the concept of the osteocyte’s involvement in maintaining bone quality through proper bone remodelling, which is substantially declining in skeletons of older individuals making them possibly more prone to fracture, is well-accepted. Our study on osteocyte lacunar morphology in auditory ossicles highlights that bone tissue can be altered without the need of viable osteocytes. In addition, otosclerosis is a disease, which is characterized by abnormal bone growth in the middle ear with high bone turnover in the ossicles leading to conductive hearing loss^28,29^. Although the causality for pathological bone growth in ossicles has not been described, misdirected osteocyte apoptosis with subsequent failure to prevent excessive bone remodelling might play a role in otosclerosis^28^. As impaired vibration of the ossicles was found in osteopetrotic mice^30^, and the deficiency of the osteocyte-secreted fibroblast growth factor 23 (FGF23) has been associated with ossicles malformation^31^, it is most likely that the well-known and bone-specific regulatory pathways are present in the ossicles.

Taken together, the pronounced osteocyte death in the auditory ossicles implicates new insights in the pathogenesis of bone loss syndromes regarding osteocyte apoptosis, since osteocyte activity influences the balance between bone formation and resorption^32^. Namely, in the ossicles, the co-occurrence of dying osteocytes and maintained structural integrity represent a unique feature. Indeed, lack of bone remodelling conserves the architecture of the auditory ossicles, which is of high importance for consistent sound transmission. Further studies are needed to investigate the exact chemical nature of inhibitor of bone resorption, i.e., by immunohistochemistry. Moreover, it has not been clear whether micropetrosis is exclusively associated with diseased bone and fracture risk, or it may also display structure-preserving effects due to stoppage of bone resorption. For this purpose, it is now required to analyze the ossicles of other mammalian species, also to put our findings in an evolutionary context. Furthermore, the analysis of immobilized bones at other skeletal sites may help in understanding whether the observed processes are a result of an absence of high impact loads, aging or rather the exclusive anatomy of the ossicles that keeps them adapted optimally to the sound transmission function.

## Methods

### Specimens

The auditory ossicles were obtained from 22 individuals following autopsy. The age of the included subjects ranged from 0 (newborns) until 86 years. Altogether, the malleus and incus of 18 patients were prepared, while the stapes was obtained from 13 patients, considering that the preparation method sometimes did not allow extracting all three ossicles from the middle ear in intact condition. Autopsy allowed identification of diseases that may have affected ossicles’ structure and composition. This way, cases afflicted with otosclerosis, cancer, renal diseases, primary hyperparathyroidism, and Paget’s disease of bone were not included in the study. Informed consent was obtained from the family members after comprehensive information on all related issues. The study was approved by the Ethics Committee of the Hamburg Chamber of Physicians (WF-70/16) and the methods were carried out in accordance with the approved guidelines.

### Micro-computed tomography (µ-CT)

To determine age-dependent changes in size and shape of the auditory ossicles, available malleus, incus and stapes were imaged with µ-CT. The specimens were scanned with a Bruker Skyscan 1272 high-resolution µ-CT system (Bruker, Kontich, Belgium) at a resolution of 6 µm at 90 kV and 111 µA with a 0.5 mm aluminium filter.

### Histology

All specimens were fixed within 24 hours in PBS buffered 3.7% formaldehyde for 3 days, dehydrated and embedded undecalcified in methyl-methacrylate following standard protocols. Subsequently, they were cut on a Microtec rotation microtome (Techno-Med GmbH, Munich, Germany). Sections of 4 µm were stained by three different staining protocols: toluidine blue, trichrome Goldner, and von Kossa-van Gieson stain. Total length of the ossicles was measured in standardized locations (Fig. 1C). The number of osteocytes per bone area (N.Ot/B.Ar, #/mm^2^) was quantified in toluidine blue stained sections. The pattern of the collagen fibres was visualized by polarized light microscopy on toluidine blue stained sections in order to differentiate between woven and lamellar bone structure. Furthermore, the thickness of the anterior crus of the stapes was measured in von Kossa-van Gieson staining. The embedded bone was further used for quantitative backscattered electron microscopy (*qBEI*).

### Quantitative backscattered electron imaging (qBEI)

Quantitative backscattered electron imaging was used to assess the degree of the mineralization of the specimens (CaMean, wt%), as well as the number of osteocyte lacunae (N.Ot.Lc/B.Ar, #/mm^2^), lacunar area (Lc.Ar, µm^2^) and the number of mineralized lacunae (N.Mn.Lc/B.Ar, #/mm^2^). The method has been previously described in detail^6,9,21,33,34^. Briefly, the scanning electron microscope (LEO 435 VP; LEO Electron Microscopy Ltd., Cambridge, England) was operated at 20 kV and 680 pA at a constant working distance (BSE Detector, Type 202; K.E. Developments Ltd., Cambridge, England). The generated grey values correlate with the calcium content (CaMean, wt%) of the cross-sectioned bone^35^. Images were thresholded with ImageJ analysis software (ImageJ 1.42; National Institutes of Health, Bethesda, MD, USA; https://imagej.nih.gov/ij/). Four images per specimen were obtained and analysed (200x magnification).

### Acid Etching and Scanning Electron Microscopy (SEM)

To visualize osteocyte lacunae and their canalicular network and quantify the number of canaliculi per lacuna (N.Ot.Ca/Ot.Lc) and the mean canalicular length, we performed acid etching on four malleus specimens belonging to different ages (0, 0.5, 22, 60 years). The embedded specimens were polished using an automatic grinding system (Exakt, Germany) to achieve a flat coplanar surface. The acid etching procedure was performed as established in our previous studies^36,37^. Namely, polished samples were submerged in 9% phosphoric acid for 20 seconds (polished side upwards) followed by a short rinse in deionized water (1–2 s). Then, they were put into 5% sodium hypochlorite for 5 minutes and rinsed in deionized water. After drying at room temperature, the specimens were sputter coated with a gold alloy and placed in the scanning electron microscope.

### TUNEL

Osteocyte apoptosis was assessed using Terminal deoxynucleotidyl transferase dUTP nick end labelling (TUNEL) assay (Roche, #116847959109) according to the manufacturer’s instructions. The malleus of each of the four age groups 0, 0.5, 22 and 60 years was decalcified, stained with TUNEL and examined by fluorescence light microscopy with FITC (Fluorescein isothiocyanate) and DAPI (4’,6-diamidino-2-phenylindole) filter. Fragmentation of DNA was analysed by TUNEL^38^ and expressed as percentage of apoptotic cells (TUNEL-positive vs. total DAPI-labelled osteocytes) counting approximately 100–120 cells per sample. Osteocyte apoptosis was further verified by visual nuclear fragmentation and blebbing using DAPI staining^39^.

### Reference point indentation

For reference point indentation (RPI), 20 plane-parallel ground, embedded mallei from qBEI were re-polished. After polishing, each specimen was mounted to a BioDent h_fc_ Reference Point Indentation instrument (Active Life Scientific Inc., Santa Barbara, CA, USA) equipped with a BP2 probe (Active Life Scientific Inc., Santa Barbara, CA, USA) as described previously by our group^9^. Prior to indentation, images were taken using opto-digital microscopy (DSX 500, Olympus, Japan) to define the correct indentation region (i.e., head to neck region of the malleus) and to avoid indentations of the PMMA filled cavities. Calibration of the indenter was performed using a polished plane-parallel PMMA block according to the manufacturer’s instructions. Here we performed RPI at a force of 6 N with an indentation frequency of 2 Hz and 10 successive indentations. The load displacement curves were recorded by the provided Biodent Software (Active Life Scientific Inc., Santa Barbara, CA, USA). For each specimen at least seven indents were performed. After the indentation procedure the indents were imaged again with the DSX 500 microscope to check whether the indents were exclusively located on mineralized hard tissue. From the load-displacement curve the following parameters were extracted:

- first cycle indentation distance (ID 1^st^, µm) – indentation depth after the first indentation cycle, which might inversely correlate with the bone material’s micro-hardness^40^
- total indentation distance (TID, µm) – the total depth of the indentation after the last cycle
- indentation distance increase (IDI, µm) – the difference between the depth of the first and last indentations
- average loading slope (Avg LS, N/µm)– the average slope of ten cycles of the load curve at the region of 50% to 100% maximum indentation force
- average unloading slope (Avg US, N/µm) – the average slope of the unloading curve of all cycles at 95% to 40% of the maximum indentation force. Average unloading slope is related to the materials stiffness^25^.

### Statistical analysis

Statistical software SPSS was used for all the analyses and p-values of <0.05 were considered significant. The age dependence of the measured parameters in the ossicles was determined using regression analysis with various models for curve estimation to find the fit that best explains age-related variability. Bivariate correlation analysis was performed to estimate the mutual correlation between various measured parameters. The data obtained for the malleus was further divided into two age groups: young (<25 years) and old (>60 years). Furthermore, malleus was divided to two zones, one being the peripheral (<200 µm from the periosteal bone surface) and the other central (<200 µm from the central blood vessel). ANOVA for repeated measurements was performed to compare the mineralization level between the central and peripheral zone of the malleus in relation to age group.

## Electronic supplementary material

Supplementary File
