# Supplementary material for: Early bone tissue aging in human auditory ossicles is accompanied by excessive hypermineralization, osteocyte death and micropetrosis
Source: Sci Rep. 2018 Jan 30;8:1920. doi: 10.1038/s41598-018-19803-2 (PMC5789841; doi:10.1038/s41598-018-19803-2)
Supplement: Supplementary file 1 — Supplementary File [file 41598_2018_19803_MOESM1_ESM.pdf]

Supplementary Figures:

“Early bone tissue aging in human auditory ossicles is accompanied by excessive hypermineralisation, osteocyte death and micropetrosis” by Tim Rolvien, Felix N. Schmidt, Petar Milovanovic, Katharina Jähn, Christoph Riedel, Sebastian Butscheidt, Klaus Püschel, Anke Jeschke, Michael Amling, and Björn Busse

Supplementary Figure 1

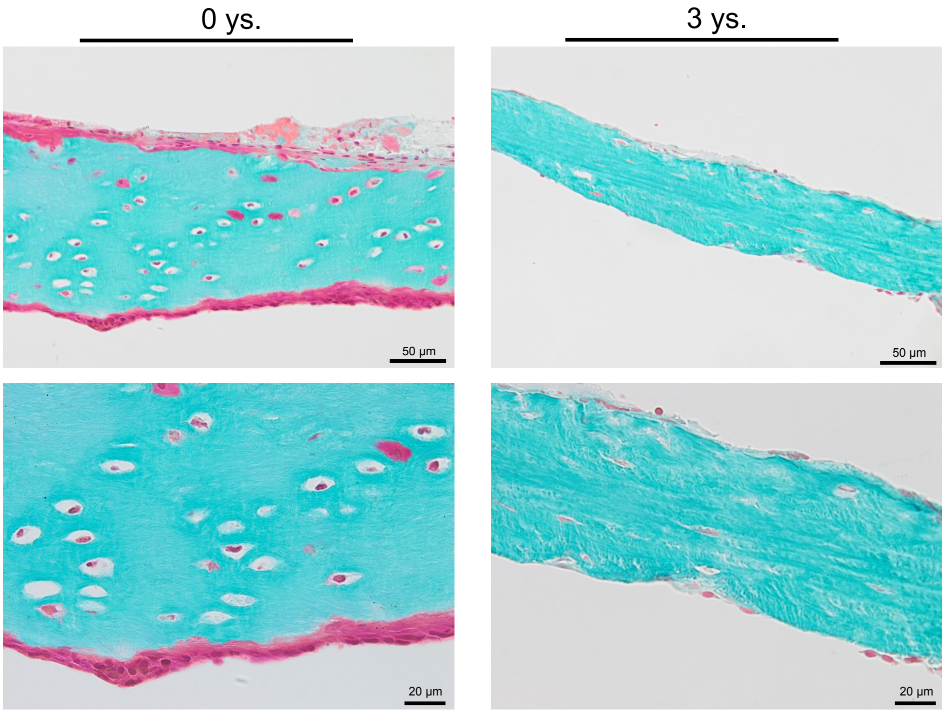

Supplementary Figure 1. Goldner Trichrome stainings of the stapes at 0 and 3 years of age. Note the highly dynamic osteocyte death within the first years of life.

Supplementary Figure 2

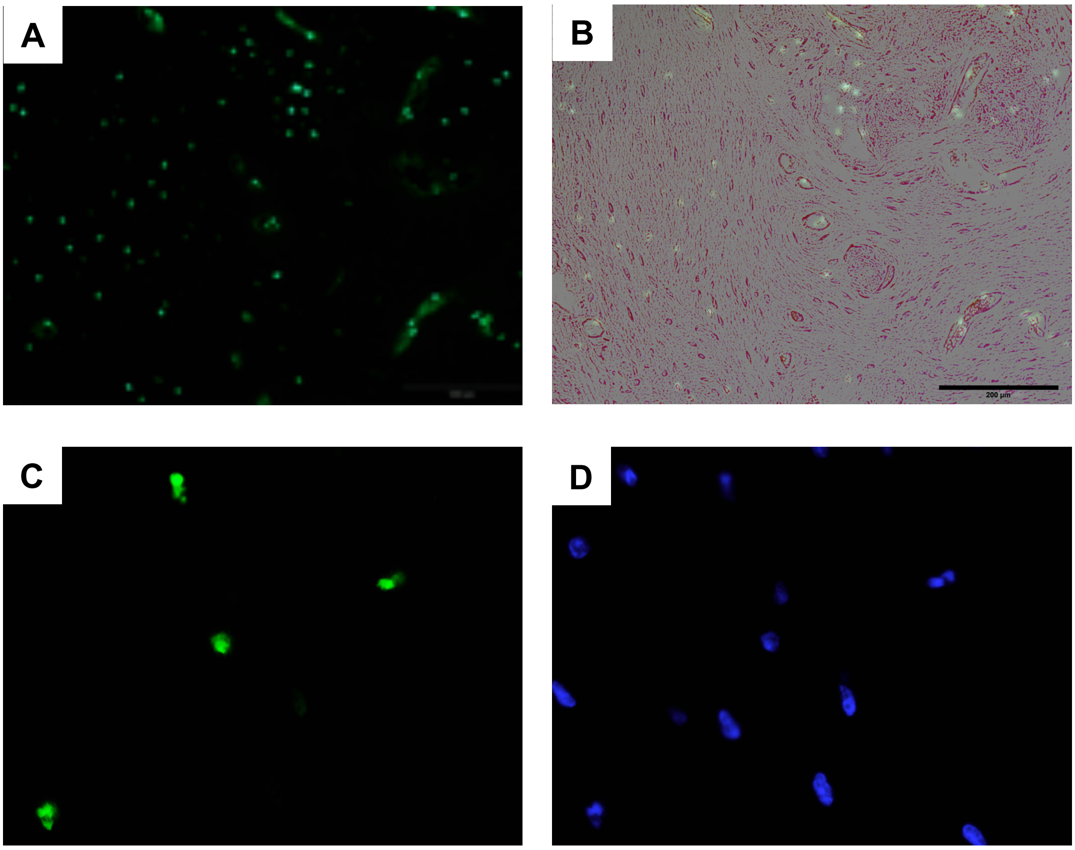

Supplementary Figure 2. Detection of apoptotic osteocytes using TUNEL. **(A, B)** TUNEL positive cells (left panel) compared to correspondent bright field images (right panel), magnification 50x. **(C, D)** Nuclear morphology and apoptotic blebbing in FITC (left panel) vs. DAPI (right panel) filter. Magnification 400x.
